# Neural–immune interactions in brain metastases from angiosarcoma: relevance of neurotransmitter-driven macrophage modulation

**DOI:** 10.1097/MS9.0000000000004867

**Published:** 2026-03-31

**Authors:** Rana Anas, Hufsa Malik, Mahnoor Fatima

**Affiliations:** a Department of Medicine, Sargodha Medical College, Sargodha, Pakistan; b Department of Medicine, Shaheed Ziaur Rahman Medical College, Rajshahi Medical University, Bogura, Bangladesh

*Dear Editor,*

Brain metastases arising from angiosarcoma remain uncommon but clinically challenging, as current systemic options offer modest benefit in most cases[1]. Their microenvironment is enriched with tumor-associated macrophages (TAMs), which have increasingly been recognized for their contribution to local immune suppression and treatment resistance^[^2,3^]^.

Within the central nervous system (CNS), macrophages exist in an environment that is continuously exposed to neurotransmitters such as catecholamines and glutamate. This neurochemical background appears to influence macrophage polarization, cytokine expression, and functional behavior^[^4,5^]^. Of note, adrenergic signaling has been linked to an M2-skewed TAM phenotype in experimental settings^[^4^]^.

Although immune checkpoint inhibitors have shown activity in angiosarcoma, responses remain variable[6], and myeloid-dominant CNS niches have been associated with reduced immunotherapy benefit across malignancies possessing brain involvement[3]. Taken together, these observations suggest that a neural–immune axis may be relevant in shaping the TAM phenotype in angiosarcoma brain metastases. Rather than implying a therapeutic strategy at present, this notion highlights a mechanistic gap that warrants further clarification.

A better understanding of this axis could help explain discrepant immunotherapy responses observed in CNS-involved angiosarcoma and may refine ongoing efforts in translational modeling. This letter is in line with the TITAN Guidelines on the need for transparency in AI use in health care[7].

## Data Availability

No data were used for this study.
